# Estimation of malaria incidence in northern Namibia in 2009 using Bayesian conditional-autoregressive spatial–temporal models^☆^

**DOI:** 10.1016/j.sste.2013.09.001

**Published:** 2013-12

**Authors:** Victor A. Alegana, Peter M. Atkinson, Jim A. Wright, Richard Kamwi, Petrina Uusiku, Stark Katokele, Robert W. Snow, Abdisalan M. Noor

**Affiliations:** a Malaria Public Health Department, KEMRI-Wellcome Trust-University of Oxford Collaborative Programme, P.O. Box 43640, 00100 GPO Nairobi, Kenya; b Centre for Geographical Health Research, Geography and Environment, University of Southampton, Highfield, Southampton SO17 1BJ, UK; c Directorate of Special Programmes, National Vector-borne Diseases Control Programme, Ministry of health and Social Services, Windhoek, Namibia; d Centre for Tropical Medicine, Nuffield Department of Clinical Medicine, University of Oxford, CCVTM, Oxford OX3 7LJ, UK

**Keywords:** ACD, active case detection, CAR, conditional auto-regressive, CPO, conditional predictive ordinate, DIC, deviance information criterion, ESRI, Environmental System Research Institute, EVI, enhanced vegetation index, GF, Gaussian field, GIS, geographic information system, GMRF, Gaussian markov random field, GPS, global positioning system, GRUMP, Global Rural and Urban Mapping Project, HMIS, Health Management Information System, INLA, Integrated Nested Laplace Approximation, JAXA, Japan Aerospace Exploration Agency, MAUP, Modifiable Areal Unit Problem, MCMC, Markov Chain Monte Carlo, MODIS, MODerate-resolution Imaging Spectro-radiometer, MoHSS, Ministry of Health and Social Services, NASA, National Aeronautics and Space Administration, NVBDCP, National Vector-Borne and Disease Control Programme, PCD, passive case detection, PHS, public health sector, RDT, Rapid Diagnostic Test, SPA, Service Provision Assessments, TRMM, Tropical Rainfall Measuring Mission, TSI, temperature suitability index, WHO, World Health Organisation, ZIP, Zero-Inflated Poisson, Namibia, Malaria, Spatio-temporal, Conditional-autoregressive

## Abstract

As malaria transmission declines, it becomes increasingly important to monitor changes in malaria incidence rather than prevalence. Here, a spatio-temporal model was used to identify constituencies with high malaria incidence to guide malaria control. Malaria cases were assembled across all age groups along with several environmental covariates. A Bayesian conditional-autoregressive model was used to model the spatial and temporal variation of incidence after adjusting for test positivity rates and health facility utilisation. Of the 144,744 malaria cases recorded in Namibia in 2009, 134,851 were suspected and 9893 were parasitologically confirmed. The mean annual incidence based on the Bayesian model predictions was 13 cases per 1000 population with the highest incidence predicted for constituencies bordering Angola and Zambia. The smoothed maps of incidence highlight trends in disease incidence. For Namibia, the 2009 maps provide a baseline for monitoring the targets of pre-elimination.

## Highlights

• Incidence is modelled using HMIS data in Namibia.
• Assembled data include parasitological and clinical diagnosed case. The clinical cases are adjusted using slide positivity rates at each facility.
• Denominator catchment population adjusted for probability for seeking treatment when sick with fever.
• Bayesian spatio-temporal model was implemented at facility level, adjusting for missing data using INLA.
• Spatio-temporal monthly maps of incidence are produced and a mean prediction for 2009 for Namibia.

## 1 Introduction

Maps of malaria transmission intensity are increasingly being used for planning, monitoring and evaluation, and resource allocation (Hay et al., 2009; Noor et al., 2010; Omumbo et al., 2013). In countries where malaria elimination is feasible, the World Health Organisation (WHO) proposes a transition from measuring risk by malaria prevalence surveys to surveillance through a combination of routine health management information systems (HMIS) and active case detection (World Health Organizastion, 2007). The year 2009 has a special significance for the fight against malaria in Namibia. This is when the Elimination Eight (E8) initiative was launched, under which eight southern African countries decided to collaborate to eliminate malaria in Namibia, Botswana, South Africa and Swaziland. Under this initiative, Namibia formally declared the ambition to eliminate malaria by 2020 (Noor et al., 2013a,b; Southern Africa Roll Back Malaria Network (Sarn), 2010). These ambitions were motivated by reported substantial declines in malaria burden in the four eliminating countries and by the 2008 global call for malaria elimination (World health Organizastion, 2008). A Namibian malaria indicator survey (MIS) conducted in 2009 showed a mean community *Plasmodium falciparum* prevalence of approximately 3% nationally (Ministry of Health and Social Services, 2010b). This is a threshold at which countries are advised to use case incidence data for measuring malaria risk (Hay et al., 2008; Yekutiel, 1960). In 2010, Namibia launched a national malaria strategy for the period 2010–2016 (Ministry of Health and Social Services, 2010c). The aim was to reduce malaria case incidence to 10 persons per 1000 population by 2013 and to move the country to pre-elimination status by 2016 where case incidence will be less than 1 person per 1000 population (Ministry of Health and Social Services, 2010c, d).

Most malaria eliminating countries in Africa, including Namibia, are yet to adopt active case-detection (ACD) systems (World Health Organization, 2012) and the main source of data for measuring disease incidence is from passive case detection (PCD), assembled through the public health sector (PHS). Such data, however, have deficiencies that limit their use for estimating overall case incidence accurately. A substantial proportion of malaria cases are treated outside of the PHS (Cibulskis et al., 2011; Cibulskis et al., 2007), while only a proportion of health facilities in the PHS submit returns and even fewer report every month of the year, making the data incomplete spatially and temporally (Gething et al., 2008; Gething et al., 2006; Murray et al., 2004; Stansfield, 2005). Third, only a subset of reported cases is diagnosed parasitologically and most of these cases are fevers that have been diagnosed presumptively as malaria (Cibulskis et al., 2011; Cibulskis et al., 2007). The use of such data therefore requires approaches that adjust for the non-utilisation of the PHS, incomplete data reporting which underestimate burden and the presumptive diagnosis which inflate incidence (Alegana et al., 2012; Cibulskis et al., 2011). In addition, these approaches must harness the spatial and temporal autocorrelation of the available data to predict at locations and periods where data are missing as well as estimate robustly the uncertainties of these predictions (Loha and Lindtjorn, 2010; Reid et al., 2012).

Bayesian hierarchical conditional auto-regressive (CAR) models can improve the quality of HMIS data at a national level, where routine surveillance is inefficient, by representing risk via a set of environmental or ecological factors and random effects using CAR priors (Barnerjee et al., 2004; Gelfand and Vounatsou, 2003; Gething et al., 2006). Examples of such approaches have been used previously in modelling spatial–temporal variation of disease risk in Yunnan province in China (Clements et al., 2009) and in identifying social and ecological factors driving malaria risk in Vietnam (Manh et al., 2011). These methods handle uncertainty in a coherent manner, are able to predict risk in areas where data are not recorded while at the same time smoothing variability where the denominator (population) is small (Gelfand and Vounatsou, 2003; Reid et al., 2012). These approaches are used in this study with the primary aim of predicting malaria incidence at second administrative unit level (constituencies) in northern Namibia where malaria is considered endemic (Ministry of Health and Social Services, 2010c). In addition, a novel approach is used to adjust PHS utilisation rates to estimate catchment population. Secondary aims of this study were to calculate populations at risk to determine areas where interventions can be targeted to provide universal coverage and to evaluate the use of environmental factors such as rainfall and vegetation indices in predicting incidence.

## 2 Methods

### 2.1 Study area

**Fig. 1:**
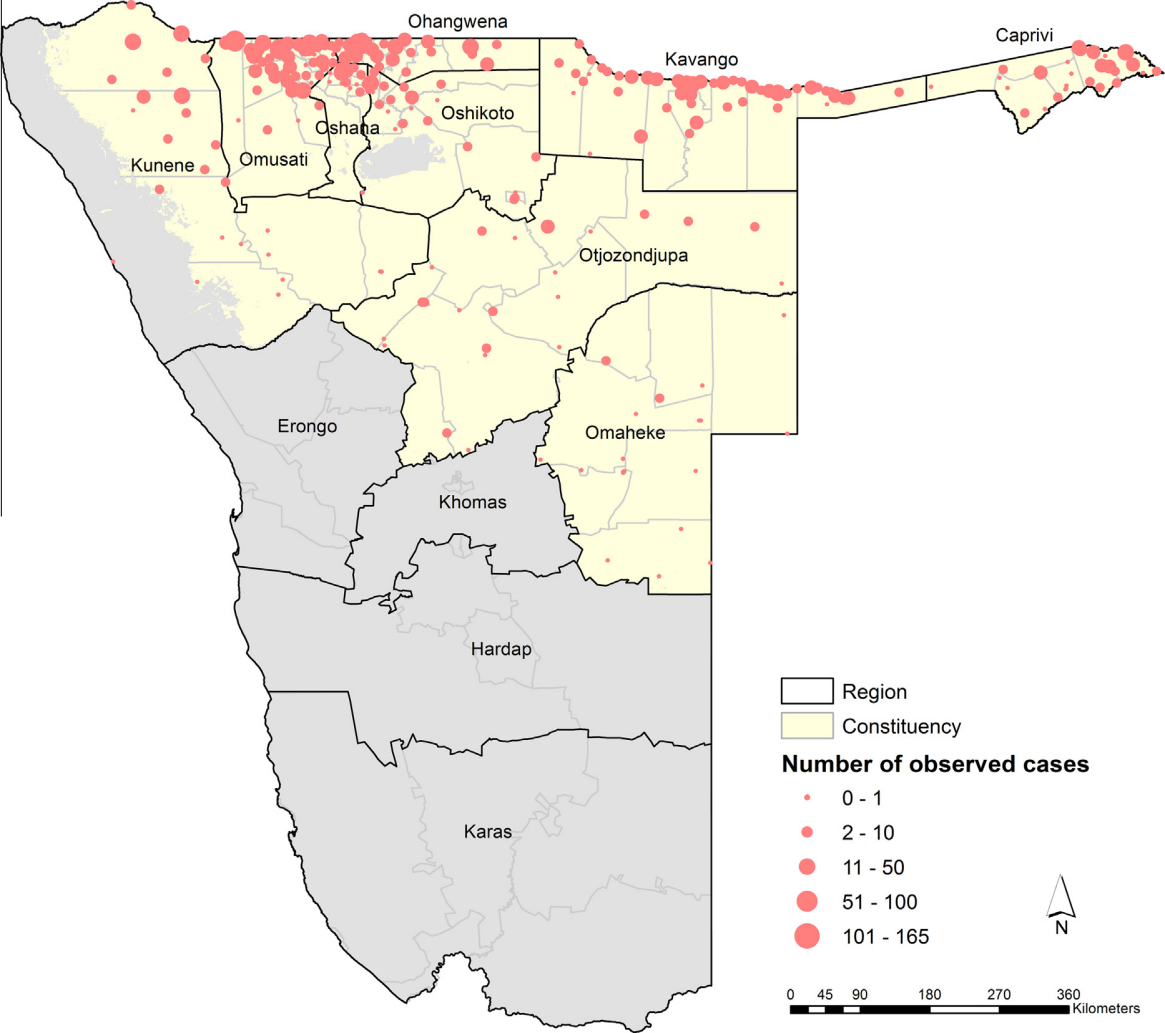
Map showing the number of cases observed at a public health facility superimposed on the 78 constituency boundaries (Administrative level 2) in the northern regions (Administrative level 1) of Namibia in 2009. The four southern regions namely Erongo, Khomas, Hadarp and Karas are considered as ‘*malaria free*’ while the grey areas in the north correspond to desert arid areas where the MODIS-derived enhanced vegetation index (EVI) was <0.1 and were, thus, considered unsuitable for transmission and masked out (Scharlemann et al., 2008). (For interpretation of the references to colour in this figure legend, the reader is referred to the web version of this article.)

Namibia is divided into 13 regions (administrative level 1) and 108 constituencies (Ministry of Health and Social Services, 2010c; Zere et al., 2006) (Fig. 1). The country is largely dry and sparsely populated with an estimated 2.1 million people in 2009 living in an area of approximately 0.83 million km^2^ (National Planning Commission, 2012). The risk of malaria is constrained by aridity (Ministry of Health and Social Services, 2010c; Snow et al., 2010) with the larger and sparsely populated south made up of four regions, Karas, Hardap, Khomas and Erongo, considered either malaria-free or supporting high focal very low transmission intensity (Ministry of Health and Social Services, 1995, 2010c). The majority of the population resides in the other nine northern regions of the country that are also considered to contribute almost the entire malaria burden in Namibia (Ministry of Health and Social Services, 2010b, c, e). In this study, analysis of malaria incidence was restricted to the 78 constituencies in the nine northern regions (Fig. 1).

### 2.2 Assembly of malaria case data

Monthly data (January to December) for 2009 on confirmed and suspected (clinically diagnosed) cases of malaria among patients of all ages were obtained from the Ministry of Health and Social Services (MoHSS) after a national Service Provision Assessment (SPA) survey was conducted (Ministry of Health and Social Services (MoHSS) and Icf Macro, 2010). The health facility survey covered 273 facilities in the north comprising of hospitals, health centres, clinics and sick bays that are managed by the Ministry of Health and Social Services (MoHSS), missions, Non-Governmental Organisations (NGOs), the private sector and Ministry of Defence (MoD) and police. Of these, only 13 were private health facilities, all located in urban centers. Three constituencies had no facility data and were treated as missing data. During the survey, a health system questionnaire was used to collect data on suspected and confirmed malaria cases for a 12-month period from patient registers. Each facility was also geo-located using a handheld global positioning system (GPS) device. Rapid Diagnostic Tests (RDTs) were used to examine blood samples from most patients at primary health facilities although a few, mostly at tertiary facilities, were examined using microscopy (Ministry of Health and Social Services, 2010a).

### 2.3 Assembling data on environmental predictors of incidence

The incidence of malaria is usually a function of its underlying transmission intensity (Patil et al., 2009) which in turn is driven by factors such as rainfall, temperature and human habitation that influence the development and survival of the malaria parasite and vector (Molineaux, 1988). The annual mean enhanced vegetation index (EVI) for 2009 derived from MODerate-resolution Imaging Spectroradiometer (MODIS) sensor imagery was used as a measure of vegetation cover (Scharlemann et al., 2008). Monthly 2009 precipitation data were obtained from the Tropical Rainfall Measuring Mission (TRMM 3B43) [http://trmm.gsfc.nasa.gov/], a joint collaboration between NASA and the Japan Aerospace Exploration Agency (JAXA) (Huffman and Bolvin, 2011; NASA, 2011). TRMM 3B43 [http://trmm.gsfc.nasa.gov/] is a gridded mean monthly precipitation product in mm h^−1^ at 0.25° × 0.25° spatial resolution (Huffman, 1997). It is produced after TRMM multi-satellite precipitation analysis (TMPA) (Huffman and Bolvin, 2011) that combines both satellite sensor data and observations from at least 6700 rain gauges from global reports and country-specific reports. A 1 km × 1 km surface depicting a temperature suitability index (TSI) for malaria transmission (Gething et al., 2011) ranging from 0 (not suitable) to 1 (most suitable) was also obtained from the Malaria Atlas Project [http://www.map.ox.ac.uk]. The annual mean values of EVI, precipitation and TSI were computed for each constituency. Finally, the proportion of urban population within each constituency was extracted based on urban extent from the Global Rural Urban Mapping Project (GRUMP) (Balk et al., 2004, Center for International Earth Science Information Network (CIESIN), 2004) overlaid on a 100 m × 100 m resolution population surface developed by Afripop (Balk et al., 2004, Center for International Earth Science Information Network (CIESIN), 2004) and available at [http://www.afripop.org/]. The assembled covariates were re-sampled to 1 × 1 km spatial resolution and a value extracted for each facility in ArcGIS 10 (ESRI, Redlands, CA, USA).

### 2.4 Analysis

#### 2.4.1 Adjusting observed malaria cases based on test positivity rates and PHS utilisation

The calculation of malaria incidence requires accurate estimates of both the number of parasitologically confirmed positive cases and the size of the population from which the cases originate. The malaria cases were computed as the sum of the parasitologically diagnosed cases presented at public sector health facilities and the suspected (clinical) cases adjusted using the *P. falciparum* positivity (microscopy or RDT) rate per facility. The focus was on the public sector, which constituted majority of surveyed health facilities (96%) and is mainly sponsored by the government and public resources. To define the catchment population two factors were considered: (a) only a subset of the population was likely to use the public health sector and; (b) these would vary geographically within a catchment area and by constituency. In Namibia, the MIS of 2009 recorded treatment seeking behaviour for fevers and showed that only 52% of all individuals who had a fever in the last 2 weeks sought treatment in the public health sector and the utilisation rate varied by region (Ministry of Health and Social Services, 2010b). To define public health facility catchment populations empirically, the treatment seeking data from the MIS were used subsequently to develop a utilisation model that defined, at every 1 km × 1 km grid cell, the probability that a febrile individual will use a public health facility using a three-parameter logistic regression model (Alegana et al., 2012). These probabilities were applied to a population surface of similar resolution (Afripop, 2010) to estimate the 2009 population seeking treatment for fever at public health sector facilities. The adjusted population counts were then used in modelling incidence.

#### 2.4.2 Preliminary analysis of environmental covariates

A non-spatial Poisson regression model was used to test the univariate and multivariate associations of assembled environmental covariates and crude incidence in R version 2.15.2 [http://www.r-project.org/]. The environmental covariates were used in the continuous form in a generalized linear regression model with the response variable being the observed crude incidence rates assuming that the expected cases have a Poisson distribution; *Y_ij_* ∼ Poisson *μ_ij_* for the *i*th observation in facility *j*. Wald’s *P*-values and goodness-of-fit statistics with associated confidence intervals were assessed. Variables significant at a *P*-value of <0.05 were selected for inclusion into the predictive model.

#### 2.4.3 Bayesian space–time zero-inflated CAR model for malaria incidence

Environmental covariates selected via the preliminary analysis, the reported cases and catchment population per public sector health facility were used in a Bayesian spatio-temporal zero-inflated conditional autoregressive (CAR) model using Integrated Nested Laplace Approximation (INLA) (Martins et al., 2013; Rue et al., 2009) to predict incidence at the constituency level. A Zero-Inflated Poisson (ZIP) model was used following the example of studies in low transmission settings, to handle count data with a lot of structural or excess zeros (Lambert, 1992; Manh et al., 2011). In Namibia, no malaria cases were reported in 65.3% of the facility level monthly returns, with 43% of facilities reporting no cases in March and over 60% from May to December. The ZIP models have also been applied previously in mapping the malaria vector sporozoite rate (Amek et al., 2011; Nobre et al., 2005) as well as in schistosomiasis (Vounatsou et al., 2009), but with inference made using the Markov Chain Monte Carlo (MCMC) approach. In this study, however, inference was made using INLA via the Gaussian Markov Random Field (GMRF) (Rue and Martino, 2007) that reduces computation time significantly (Kneib et al., 2010; Rue et al., 2009; Rue and Martino, 2007). In addition, a facility random effect model was fitted to allow for variation between two or more facilities in the same constituency. For the ZIP model, the probability of observing zero (Böhning, 1998; Ghosh et al., 2004; Neelon et al., 2010) is;

$$P_{\mathrm{ij}}(y_{i}=0)=(1-P_{\mathrm{ij}})+P_{\mathrm{ij}}e^{-\mu_{\mathrm{ij}}}\;0\leq p\leq 1$$

$$P_{\mathrm{ij}}(y_{i}=k)=P_{\mathrm{ij}}\frac{\mu_{\mathrm{ij}}^{k}e^{-\mu_{\mathrm{ij}}}}{k!}\;k=1\text{,}\ldots \text{,}\infty$$

The term (1*−P_ij_*) in the first part represents the probability of observing a true zero and, therefore, when *P* = 1 the equation reduces to a general Poisson model and zero is inflated when *P* < 1. Covariates were introduced via a log linear model for *μ_ij_* while maps of predicted monthly and annual incidence were produced at constituency level. In the model, the observed variables *y_i_*, *i *= 1, …, *n* and the linear predictor *η_i_* were modelled with additive effects as (Rue et al., 2009; Rue and Martino, 2007; Schrödle and Held, 2010):

$$\eta_{i}=\alpha +\overset{\mathrm{nf}}{\underset{j=1}{\sum }}f^{\left(j\right)}(u_{\mathrm{ji}})+\overset{n\beta }{\underset{k=1}{\sum }}\beta_{k}z_{\mathrm{ki}}+\varepsilon_{i}$$

where, *f*^(^*^j^*^)^ is a linear function on some variables u, $\beta_{k}$ are the coefficients for the covariates *Z* and *ε* represents the unstructured effects. Rue and Martino (2007) show that the posterior marginal can be estimated as:

$$\overset{˜}{\pi }(x_{i}|y)=\underset{k}{\sum }\overset{˜}{\pi }(x_{i}|\theta_{k}\text{,}y)\overset{˜}{\pi }(\theta_{k}|y)\Delta_{k}$$

with the sum evaluated using appropriate weights $\Delta_{k}$ solved at suitable reference points $\theta_{k}$ (Rue and Martino, 2007; Schrödle and Held, 2010). The posterior marginal π(θ|y) of the hyper-parameters are evaluated as:

$$\overset{˜}{\pi }(\theta |y)\alpha \frac{\pi (x\text{,}\theta \text{,}y)}{\overset{˜}{\pi }G(x|\theta \text{,}y)}|x=x^{*}(\theta )$$

with the denominator as a Gaussian approximation of π(x|θ,y) and *x*^∗^(*θ*) being the mode of the full conditional π(x|θ,y) (Schrödle and Held, 2010). The final log-relative risk model was represented as:

$$\eta_{i}=\log(E_{i})+\mu +Z_{\mathrm{ij}}^{T}\beta_{\mathrm{ij}}+f(s_{u})+\psi_{i}+f(t)$$

with the *E_i_* being the expected number of confirmed and suspected cases adjusted for slide positivity at each facility *i*, the term μ represents the intercept, with the *f*(*s_u_*) and *f*(*t*) terms representing the spatially unstructured effects and seasonal effects, respectively. The conditional autoregressive prior *ψ_i_* was included to account for the assumption that neighboring polygons have similar incidence (Barnerjee et al., 2004). This specification ensures a smoothed map of risk with geographically reliable estimates (Bernardinelli et al., 1997; Kleinschmidt et al., 2002). Full Bayesian specifications were completed by specifying priors for the fixed effects and random components. The conditional prior for neighbouring regions $\left(\phi_{j}\text{,}j\neq i\right)$ was specified following Bernardinelli et al. (1997) as $\left(\phi_{i}∼N(\mu_{\phi i}\text{,}\sigma_{\phi i}^{2}\right)$ where $\mu_{\phi i}=\Sigma_{j\neq i}W_{\mathrm{ij}}\phi_{j}/\Sigma_{j\neq i}W_{\mathrm{ij}})$; $\sigma_{\phi i}^{2}=1/\gamma_{\phi }\Sigma_{j\neq i}W_{\mathrm{ij}})$. The *W_ij_* is the adjacency matrix of weights assigned as *W_ij_* *=* 1 for two neighbouring regions or *W_ij_* = 0 otherwise. A full treatment on CAR modelling theory can be found elsewhere (Barnerjee et al., 2004; Gelfand and Vounatsou, 2003). The random effects component was specified as a set of vague normal priors.

Two CAR models were fitted: Model 1 included a spatio-temporal component but excluded environmental covariates such as vegetation indices, whilst Model 2 included these environmental covariates in addition to spatio-temporal structure.

#### 2.4.4 Computing the cross-validation statistics and proper scoring rules

The performance of both CAR models was compared using the deviance information criterion (DIC) (Spiegelhalter et al., 2002). Predictive model assessment was conducted using the probability integral transform (PIT) and the conditional predictive ordinate (CPO), a leave-one-out cross-validation approach in which a prediction is validated based on the fitted model and the remaining data only (Czado et al., 2009; Spiegelhalter et al., 2002). The CPO, defined as the probability of observing a value given all other data, was examined for all observations in a full Laplace model (Martins et al., 2013). Both these measures assess the calibration (statistical consistency) and sharpness (concentration) of the predictive model. The predictive measures of fit have been shown to fail if the approximation of the latent Gaussian Field (GF) is not accurate (Czado et al., 2009). Model scoring rules such as the square error score (SES) and the ranked probability score (Gneiting and Raftery, 2007) as well as Pearson correlation of observed and predicted incidence were computed. The latter was based on 26 health facilities selected randomly as validation set. Proper Bayesian scoring rules are discussed by Gneiting and Raftery (2007) and implemented using a predictive distribution (Supplementary information). For example, the RPS generalizes the absolute error and is minimum for true predictions (Czado et al., 2009).

### 2.5 Population at risk

To estimate the population at risk at varying levels of malaria incidence, the total population resident in constituencies living in the six predicted endemicity classes of: less than 1; 1–5; 5–10; 10–15; 15–20 and greater than 20 cases per 1000 population was calculated. The population surface was obtained from Afripop (Afripop, 2010) which had been developed from a combination of census, population settlements and land cover by disaggregation of census data to improve their spatial resolution (Linard et al., 2010; Linard et al., 2012). The population surface had also been used in mapping health facility catchment population in Namibia (Alegana et al., 2012). The original population surface, produced for 2010 from Afripop, was back-projected to 2009 using the United Nations’ inter-censual growth rates (http://esa.un.org/unup/) and categorized according to estimated risk in northern Namibia.

## 3 Results

### 3.1 Malaria incidence and facility attendance characteristics

A summary of the assembled malaria incidence data and modeled estimates of public health sector utilization are shown by health district in Table 1. Overall, only 17 PHS facilities had no malaria reports in 2009 and the remaining health outlets returned complete reports every month. Most health facilities were located in Caprivi, Kavango, Ohangwena, Oshana and Omusati regions where population density is greatest (Table 1 and Fig. 1). The spatial distribution of reported cases, including suspected cases adjusted for test positivity rates, is shown in Fig. 1 and indicates higher caseloads in the northern regions. In total, 134,851 cases were clinically diagnosed while 90,835 individuals were examined for malaria parasites of which, 9893 were positive. The mean test positivity rate was 11.2 [95% CI 6.7–15.7] (Table 1). Crude annual incidence based on the parasitological and clinically diagnosed cases, the latter corrected for slide positivity rate, was 16 cases per 1000 population. This was highest in the first 4 months of the year and peaked in March (Fig. 4). The highest crude incidence was in constituencies in Caprivi, Ohangwena and Kunene that border Angola where test positivity rates were also highest (Table 1).

### 3.2 Preliminary model involving environmental covariates

Of the selected environmental variables, univariate non-spatial regression analysis showed that the EVI (coefficient of regression, 95% CI: 6.55, 4.25–8.87, *p* < 0.001), TSI (7.57, 5.34–9.96, *p* < 0.001) and precipitation (0.02, 0.01–0.03, *p* = 0.002) were significant predictors of crude incidence. In addition, the percentage of urban resident population produced a negative and significant association with incidence (−0.01, −0.01 to −0.00, *p* < 0.001). In the multivariate model, that included all four covariates, only EVI (14.29, 9.24–19.42, *p* < 0.001) was positively associated with crude incidence and was included in the final model. The number of environmental covariates was minimized in the final model to achieve a parsimonious space–time model and due to the observed large correlation between some covariates, for example altitude and temperature or vegetation indices and rainfall (Craig et al., 2007; Pascutto et al., 2000).

### 3.3 CAR model predictions of monthly and annual incidence for 2009

Two spatio-temporal models of incidence were implemented. Model 2 included EVI while Model 1 excluded the covariate information. Table 2 lists Bayesian model parameters for the two CAR models with and without environmental covariate. Overall, Bayesian model parameters for seasonal random effects (2.02 with Crl 0.16–5.79), facility random effects (6.95, Crl 2.65–13.22) and unstructured random effects (0.20, CrI 0.02–0.57) were all significant at 95% Crl (Bayesian credible interval). There were also marginal differences in the overall mean: −1.80 Crl (−1.98 to −1.64) and −1.76 Crl (−1.93 to −1.58) for model with and without covariate information respectively.

Table 3 compares these two models based on the DIC, which represents a trade-off between model complexity and goodness-of-fit, and SES. The EVI improved the model fit marginally, as indicated by the lower DIC for Model 2 in Table 3. The SES for M2 (1.61) was lower than that for M1 (1.70) suggesting a better predictive performance for M2 although only marginally. The conditional predictive ordinate (CPO), a cross-validation logarithmic score, was also calculated for each prediction. For both models the CPO score was 0.22 (Table 3) and since a smaller CPO value usually indicates greater predictive accuracy (Schrödle and Held, 2010), this also suggests a small difference between the two fitted models. However, in view of its lower DIC, Model 2 (with EVI) is used as the basis for presenting subsequent model outputs. The Pearson correlation coefficient for this model based on a hold out set was 0.56.

**Fig. 2:**
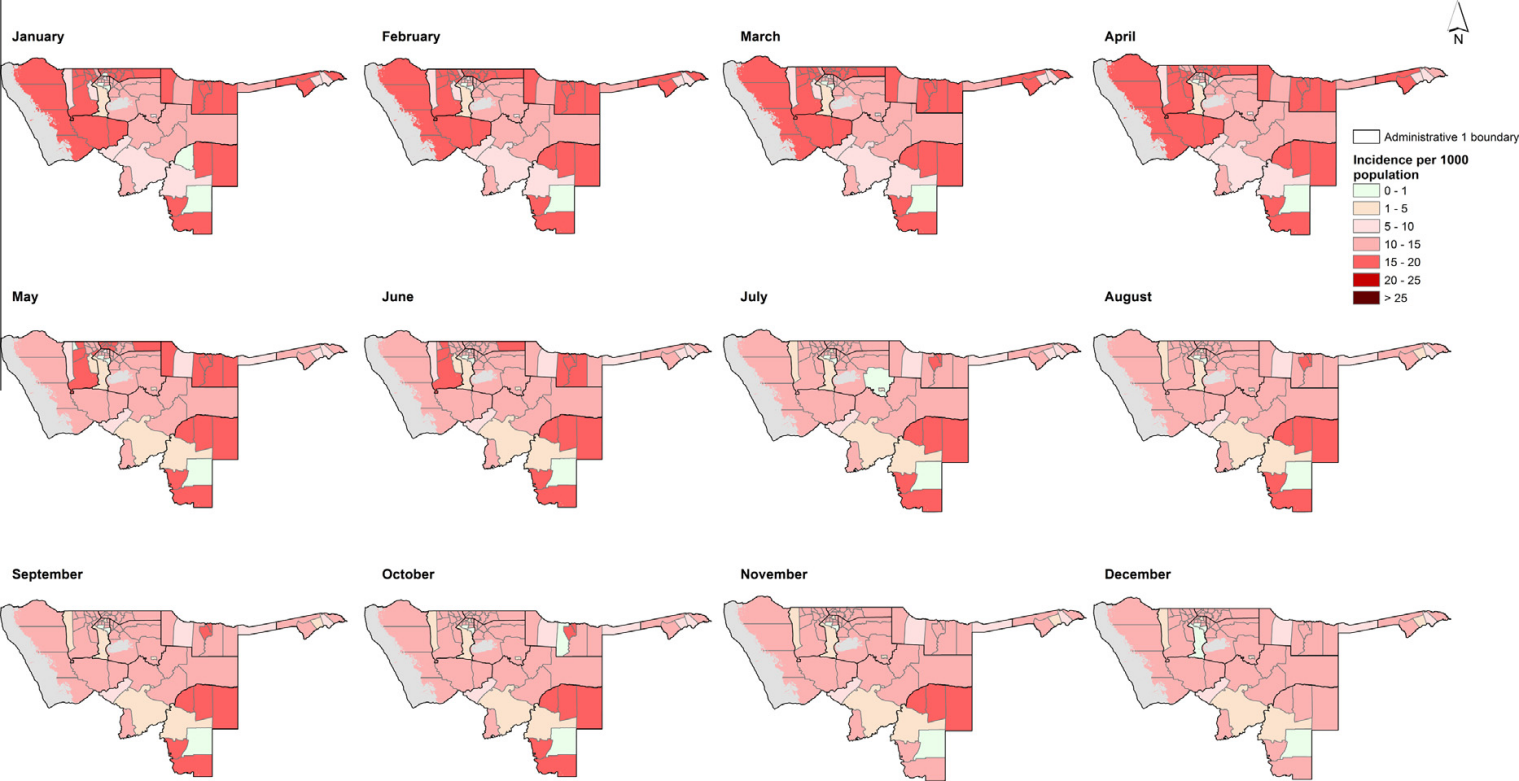
Map showing the predicted monthly malaria incidence per 1000 population at constituency level for regions in the north of Namibia in 2009 using Bayesian CAR with environmental covariates (Model 2).

**Fig. 3:**
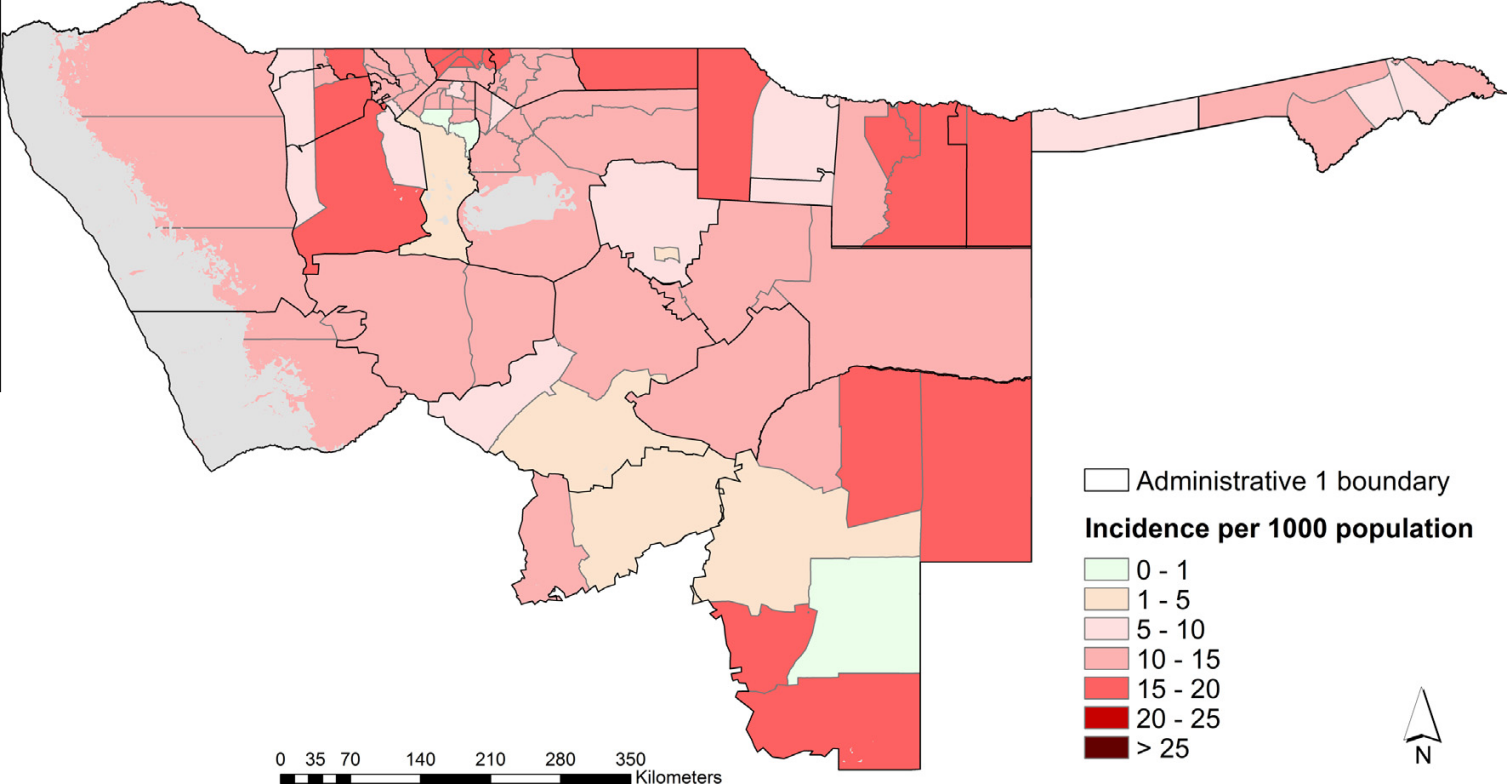
Map showing the mean annual incidence prediction based on Bayesian CAR with environmental covariates (Model 2).

**Fig. 4:**
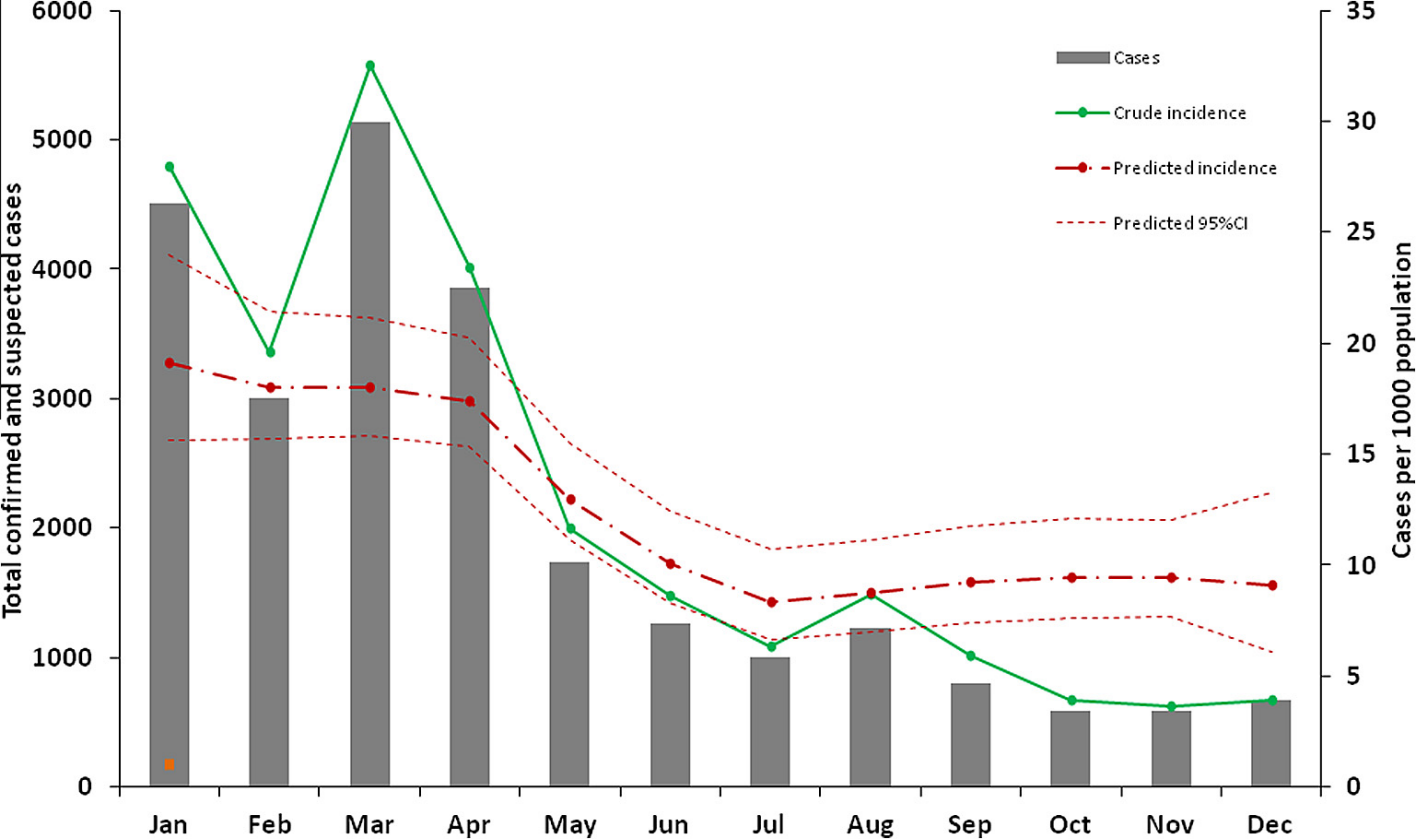
Plot of the reported cases by month in northern Namibia in 2009 (vertical dark grey bar), the calculated crude incidence (green line) derived from combined confirmed and suspected cases and the predicted incidence per 1000 population (dashed-dotted red line) with 95% Crl upper and lower limits. (For interpretation of the references to colour in this figure legend, the reader is referred to the web version of this article.)

Overall malaria incidence peaked in the months of March and April and was highest in Kunene, Kavango, Caprivi and in a few constituencies in Ohangwena region as shown in Fig. 2, based on Model 2. Fig. 3 shows a map of mean annual incidence based on this model choice. The predicted mean annual incidence of the Bayesian CAR model was 13 cases per 1000 population in the 78 constituencies in northern Namibia. The highest predictions were between 15 and 20 cases per 1000 population (Fig. 4).

### 3.4 Population at risk

Based on Model 2, 383,632 people (27.2% of the population) lived in areas where case incidence was greater than 15 cases per 1000 population; slightly more than half 745,903 (52.9%) lived in areas where case incidence was between 10 and 15 cases per 1000 population; approximately 216,512 (15.4%) resided in regions with an average of 5–10 cases per 1000 population; 49,005 (3.5%) in areas with greater than 1 case, but less than 5 cases per 1000 population and 1% of population lived in regions with less than 1 case per 1000 population. Population density was highest in the northern border constituencies.

## 4 Discussion

The evaluation of pre-elimination status requires a detailed description of local epidemiology of malaria transmission patterns. From the predicted monthly maps of Namibia (Fig. 2 and Fig. 4), a higher incidence of malaria was observed between January and April in the constituencies bordering Angola and Zambia, while, lower values were observed for the July and December period. The overall mean incidence was 13 cases per 1000 population for 2009 (Fig. 3). The model included the unstructured random component to explain unobserved effects and the inclusion of the structural effects via the GMRF introduced dependence resulting in spatial and temporal smoothing of seasonal variation (Banerjee and Carlin, 2003; Rue and Held, 2005). The Bayesian CAR approach has the advantage of addressing several sources of uncertainty. The model was applied at facility level and, therefore, the method not only takes into account the nature of the facility, but also season and environmental factors in adjusting for under-reporting. In addition, the CAR model smoothed incidence, thereby addressing the potential impact of model instability resulting from small numbers of reported cases, apparent in the facility data presented in Fig. 1. Smoothing incidence also reduces the potential impacts of under-reporting of cases by facilities. Secondly, incorporating the environmental covariate explained spatial variation where data were absent in addition to providing information on the climatic suitability of malaria transmission, for example, in Omaheke region (Craig et al., 1999; Guerra et al., 2008). This suggested that the inclusion of environmental covariates improved the model estimates for a few constituencies (in Kunene and Omaheke), but only marginally.

The mean incidence observed for 2009 was highest in constituencies in Omusati, Kavango and Omaheke region bordering Angola and Botswana. Historical *P. falciparum* data for Namibia between 1969 and 1992 (Noor et al., 2013a,b) suggest a parasite prevalence of greater than 5% in Kavango and other northern regions along the border with Angola. In addition, Craig and others showed that in Botswana, the area along the north-western border areas with Namibia had relatively high prevalence (Craig et al., 2007). For these border constituencies concerted efforts with neighbouring countries have to be put in place to realize the pre-elimination targets (Noor et al., 2013b,c). Incidence in these regions could well be driven by cross border population movement (Cosner et al., 2009). Similar suggestions were made for two districts in South Africa close to the Mozambique border (Kleinschmidt et al., 2002) and in Yunnan province in China that borders Myanmar, Laos and Vietnam (Clements et al., 2009).

The approach presented here drew upon a comparatively data rich setting and the facility census used may not be available in many countries. The recent improvements in case management in Namibia in which all suspected malaria fevers are diagnosed parasitologically before treatment will reduce the need for adjustment for test positivity rate. In addition, planned improvements in HMIS reporting and quality and transition to active case detection mean smaller adjustments for treatment seeking and reporting will be required in future. This is may also be useful for external validation, with additional resources, of approaches used in this study. These factors will, therefore, contribute to the precision of routine malaria case data in estimating disease burden in the future. More precise incidence estimates should provide a basis for targeting active case detection efforts at specific locations and in specific months, potentially making such resource-intensive efforts more cost-effective. A comparison of our predictions with the standard WHO approach shows that the latter estimates a higher annual malaria incidence of 23 per 1000 population in 2009 in Namibia and generally followed a pattern close to that of the crude incidence (Supplementary information). The WHO approach is described in detail elsewhere (Cibulskis et al., 2011). The main difference in our approach is the use of health facility as a random effect in the model and the utilization of the spatial and temporal autocorrelation in the data resulting in smoothing of the predictions.

Bayesian hierarchical models are often implemented using numerical statistical methods such as Markov Chain Monte Carlo (MCMC) and Laplace transformation amongst others (Cressie and Wikle, 2011) (p. 238). When large data set are involved, MCMC computation can be demanding and the Gaussian Markov Random Field (GMRF) (Rue and Martino, 2007) offer an alternative approach due to the sparseness of resulting covariance matrixes. Thus, they are computationally faster and with desirable Markov properties (Kneib et al., 2010; Rue et al., 2009; Rue and Martino, 2007). GMRF are implementable in INLA (Martins et al., 2013), although, the result are more accurate if the number of hyperparameters in model implemented is small typically less than 12 (Kneib et al., 2010; Rue et al., 2009; Rue and Martino, 2007).

One drawback of many studies analyzing areal data, and one common to the Bayesian approach used in this study, is the modified areal unit problem (MAUP), a well-known analytical problem in geography that could affect the observed statistical results with a change in shape or size of spatial polygons used in the analysis (Barnerjee et al., 2004; Robinson, 1950; Wakefield, 2003). In this study constituencies were selected as the basis for presenting predictions, with the aim of providing information at this level to health authorities, though the model was fitted at facility level. There is therefore a potential impact of MAUP both in terms of the shape of constituencies and in predicting at constituency level from facility level data. Secondly, the data used for this study were obtained from the Namibia HMIS which covers the majority of public health facilities in the north. This means that the findings are relevant only for the 12-month time-series in 2009. The results could be improved by inclusion of more data and at different time points to draw more stable long-term spatio-temporal patterns (Zhou et al., 2005). In addition, the modelling approach excluded the effects of population movements between regions, especially across borders, while the relations between the environmental variables could change across space and at shorter time periods than those considered (Hay et al., 2008). Finally, some sources of uncertainty remain. In particular, the underlying the care-seeking behaviour data used to adjust denominator populations relate to children under 5 years, not the whole population. Utilisation rates were estimated from cross-sectional surveys and therefore may not capture temporal changes in care-seeking behaviour. The underlying utilisation data also relate to fever rather than malaria *per se*.

## 5 Conclusion

Although Namibia faces a significant malaria case incidence in the border regions, the results of this analysis suggest that the country may be within the pre-elimination targets in most parts of the northern region. The NVDCP has initiated a process of creating a malaria-free buffer extending approximately 25 km across the border with Angola as well as with Zambia and Botswana (Ministry of Health and Social Services, 2010c; Noor et al., 2013b, Trans-Zambezi Malaria Initiative (Tzmi), 2012). This study provides additional information to identify the highest malaria risk areas in Namibia and when used together with evidence from modelled community parasite prevalence surveys on receptive and contemporary malaria risk, should support malaria control and elimination initiatives in the country.

## Competing interests

Authors declare no competing interests.

## Authors’ contributions

VAA was responsible for study design, data cleaning, analysis, interpretation, drafting and production of the final manuscript. RK and BN contributed to the data assembly, cleaning and contributed to the final manuscript. PMA and JW were responsible for analysis, interpretation and production of the final manuscript. AMN and RWS were responsible for overall scientific management, analysis, interpretation and preparation of the final manuscript. All authors read and approved the final manuscript.

## Funding

VAA is supported by a Commonwealth fellowship (KECS-2012-601). AMN is supported by the Wellcome Trust as an Intermediate Research Fellow (#095127). RWS is supported by the Wellcome Trust as Principal Research Fellow (#079080). This work was partly funded by a grant from the Namibia Ministry of Health and Social Services-Global Fund Programme and a Wellcome Trust Major Overseas Programme grant to the KEMRI/Wellcome Trust Research Programme (#092654). The funders played no role in the study design, data collection and analysis, decision to publish, or preparation of the manuscript.

## Figures and Tables

**Table 1:** Summary of malaria incidence and modeled facility attendance by administrative region and health district in northern Namibia.

Region	Health district	Number of health facilities (number with missing data)	Number of constituencies	Confirmed malaria cases	Suspected malaria cases	Mean slide positivity rate (95% CI)	Population 2009	Percent of population attending a PHF^a^ modelled
Caprivi	Katima	27(2)	6	954	10,605	21.1 (17.9–24.3)	87088	68

Kavango	Andara	10(0)	1	309	4293	9.2 (7.0–11.3)	26,677	71.1
Nankudu	11(1)	2	244	7662	8.4 (6.0–10.8)	48,715	64.2
Nyangana	8(0)	1	665	3063	25 (20.1–29.9)	19,815	71.9
Rundu	23(1)	5	1176	34,608	16.4 (13.4–19.4)	119,855	71.1

Kunene	Khorixas	8(0)	1	1	89	2.7 (-0.5–6.1)	12,469	61.4
Opuwo	14(0)	3	539	856	47.3 (40.7–53.8)	52,485	52.5
Outjo	4(0)	2	1	53	1.1 (-0.2–2.5)	20,395	53.4

Ohangwena	Eenhana	10(1)	4	379	3956	7.1 (4.8–9.4)	80,419	68.2
Engela	16(0)	6	916	13,774	9.8 (7.8–11.9)	131,744	74.2
Kongo	4(1)	1	529	1788	24.3 (15.8–32.8)	24,744	61.5
Omaheke	Gobabis	14(2)	7	11	96	13.8 (9.5–18.1)	68,433	62.1
Omusati	Okahao	9(1)	2	384	9066	4.1 (2.1–6.1)	29,964	73.6
Oshikuku	19(0)	5	436	10,315	3.6 (2.6–4.7)	101,587	75.2
Outapi	10(0)	2	1,970	9846	8.4 (6.6–10.2)	48,812	70.8
Tsandi	10(1)	3	617	5339	8.4 (6.2–10.6)	54,418	70.1

Oshana^b^	Oshakati^b^	19(4)	10	353	9133	3.1 (1.9–4.3)	169,053	75.4

Oshikoto	Onandjokwe	16(0)	8	266	8516	2.3 (1.6–3.1)	146,436	69.8
Tsumeb	5(1)	2	28	628	5.1 (1.8–8.4)	29,094	67.4

Otjozondjupa	Grootfontein	6(0)	2	59	547	13.7 (7.1–20.3)	33,347	61.3
Okahandja	2(1)	2	3	110	5.2 (0.2–10.1)	40,209	64.2
Okakarara	5(0)	1	17	189	11.6 (5.0–18.2)	21,748	56.6
Otjiwarongo	10(1)	2	36	319	5.4 (2.6–8.1)	42,336	67.3

Total		260(17)	78	9,893	134,851	11.2 (6.7–15.7)	1,409,841	65.3^c^

a PHF is an abbreviation for ‘Public Health Facility’, which in this case does not include private facilities or privates for profit.

b Two constituencies in Oshana region (Okatyali and Ompundja) did not have any health facilities, thus, the polygons where treated as missing data.

c Public health facility attendance for treatment of fever based on probability of attendance and the distance decay effect. Description outlined in Alegana et al. (2012).

**Table 2:** Parameters for two Bayesian zero-inflated CAR models of malaria incidence in northern Namibia on a log scale.

Parameter	Model 1	Model 2
Without covariates: posterior mean, median, (95% CrI^1^)	With environmental covariate: posterior mean, median, (95% CrI^1^)
*μ* (Intercept)	−1.763, −1.760 (−1.932 to −1.581)	−1.803,−1.800 (−1.980 to −1.639)
Enhanced vegetation index (EVI)	–	0.093, 0.093 (−0.028–0.211)
*ϑ* (parameter for Zero-inflation)	0.843, 0.843 (0.833–0.856)	0.843, 0.843 (0.833–0.854)
*τ_m_* (seasonal random effect)	1.546, 1.023 (0.137–4.692)	2.015, 1.427 (0.161–5.789)
*τ_f_* (facility random effect)	6.912, 5.836 (2.605–14.830)	6.952, 6.388 (2.641–13.220)
ϒ (unstructural random effect)	0.190, 0.136 (0.020–0.542)	0.200, 0.144 (0.019–0.568)
*ф* (structural random effect)	0.081, 0.045 (0.003–0.278)	0.080, 0.004 (0.030–0.276)

1 Crl is abbreviation for Bayesian credible interval.

**Table 3:** Posterior mean deviance, the number of effective parameters, the DIC and CPO score for each implemented model.

Model	Mean deviance	Number of effective parameters	DIC	CPO	SES
Model 1 (without covariate)	3113.22	9.79	3123.89	0.229	1.704
Model 2 (with covariate)	3112.08	10.68	3123.75	0.229	1.609
