# Supplementary material for: Estimation of malaria incidence in northern Namibia in 2009 using Bayesian conditional-autoregressive spatial–temporal models
Source: Spat Spatiotemporal Epidemiol. 2013 Dec;7:25–36. doi: 10.1016/j.sste.2013.09.001 (PMC3839406; doi:10.1016/j.sste.2013.09.001)
Supplement: Supplementary data [file mmc1.doc]

**Supplementary Information**

**Estimation of malaria incidence in northern Namibia in 2009 using Bayesian Conditional Auto-Regressive spatio-temporal models**

Victor A Alegana

**Author for correspondence**

Email: [valegana@nairobi.kemri-wellcome.org](mailto:valegana@nairobi.kemri-wellcome.org) or [valegana@gmail.com](mailto:valegana@gmail.com) Postal address: P.O. Box 43640-00100, Nairobi, Tel: +254 20 2715160 or 2720163 or 2719936, Fax: +254 20 2711673

**1.0 Introduction**

This supplementary information provides details of a routine method used by the WHO to estimate incidence and addition Bayesian model validation results. The objective was to provide additional comparison of the results from this approach and a sophisticated analysis applied in the main paper. Comparison of the results from these two methods is described from Section 1.2 to section 1.5. A brief discussion based on these comparisons is also provided (Section 1.6). Additional Bayesian validation results are presented in section 2.0.

**1.1 The WHO (non-spatial) approach for computing malaria incidence**

The WHO malaria incidence estimation method is described in detail elsewhere (Cibulsk*is et a*l., 2011). Briefly, the upper and lower limits of incidence were estimated as a ratio of the total number of malaria cases divided by the population using a public health facility, while at same time adjusting for reporting completeness. The formula is provided in Cibulskis et al. (2011) as:

$$Incidence_{upper}=\frac{Confirmed+spr\times Suspected}{r\times p}$$

$$Incidence_{lower}=\frac{(Confirmed+spr\times Suspected)(1-n)}{r\times p}$$

where: *spr* is the slide positivity rate; *r* is the rate of reporting by health facilities; *p* is the population that sought fever treatment in the public sector and *n* is the proportion of suspected cases that do not seek treatment. In this case, *p* and *n* are derived from household surveys such as the Malaria Indicator Survey (MIS) (Ministry of Health and Social Services, 2010), Demographic Health Survey (DHS) (Ministry of Health and Social Services, 2008) or Multiple Indicator Cluster Surveys (MICS) (UNICEF, 2012). The reporting rate at a regional level for all the facilities was calculated as the number of health reports received divided by the expected total number of reports in 2009.

Malaria cases comprised the parasitologically diagnosed cases, plus suspected (clinical) cases adjusted using the slide positivity (microscopy or RDT) rate per facility. A facility utilisation rate was derived from the proportion of population that sought treatment for fever at regional level from the Namibia malaria indicator survey (MIS) of 2009. For the lower limit estimate, the numerator was adjusted based on the regional proportions of cases not treated at a public health facility as presented in Cibulskis et al. (2011). The upper incidence in this case assumes 100% attendance while the lower incidence includes only the proportion likely to attend a health facility. The final maps presented based on this approach are the arithmetic means of the lower and upper incidence for comparison with incidence from the Bayesian approach and are subsequently used to estimate populations at risk.

**1.2 Comparison of predictions of monthly incidence for 2009 in northern Namibia from a spatio-temporal (Bayesian) approach and a non-spatial (WHO) approach.**

The monthly maps of incidence based on the WHO approach are shown in Figure 1 while Figure 2 is based on the Bayesian CAR modelling approach with environmental covariates. Figure 3 is a comparison of the predicted incidence based on the two methods, the crude incidence and the assembled malaria cases by month. In both the WHO method and Bayesian CAR methods, the areas at greatest risk were in Kunene, Kavango, Caprivi and a few constituencies in Ohangwena. The highest monthly incidence for both approaches was also observed in these regions with a peak in March and April. For some constituencies, the monthly incidence was greater than 25 cases per 1000 population using the WHO approach (Figure 1) compared to a more smoothed risk profile based on the Bayesian CAR approach in Figure 3. A test of concordance (Bland and Altman, 1986, Bland and Altman, 2010, Cox and Steichen, 2007) of incidence based on the WHO approach and predicted incidence from the Bayesian model in the constituencies in these border regions, produced a mean difference of 4.332 [CI 3.157 – 5.514] (95% limits of agreement -1.620 – 10.290). Pitman’s test (Pitman, 1939) which is a measure of correlation between differences in incidence and the averages, was 0.474 (p<0.001, Pearson’s *r* = 0.917). Graphically, these statistics correspond to a plot of difference in predicted incidence between the two methods against the arithmetic mean of the two predictions. In this context, the limits of agreement and a Pitman value close to zero suggest concordance (Bland and Altman, 2010, Cox and Steichen, 2007). For the May to December period, the concordance test yielded a mean difference of 3.087[CI 1.229 – 4.944] (95% limits of agreement -1.649 – 7.823); Pitman’s value of 0.794 (p<0.001) and Pearson’s *r* = 0.960). For constituencies in Omaheke region, where data were available only for 16 facilities, comparisons of differences and means based on same test yielded a mean difference 0.026 [CI 0.011 – 0.063] and suggested that for 95% of observations the incidence would be between 0.004 and 0.168. The Pitman’s value was, however, 0.989, *p*<0.001 and Pearson’s *r* = 0.244.

**
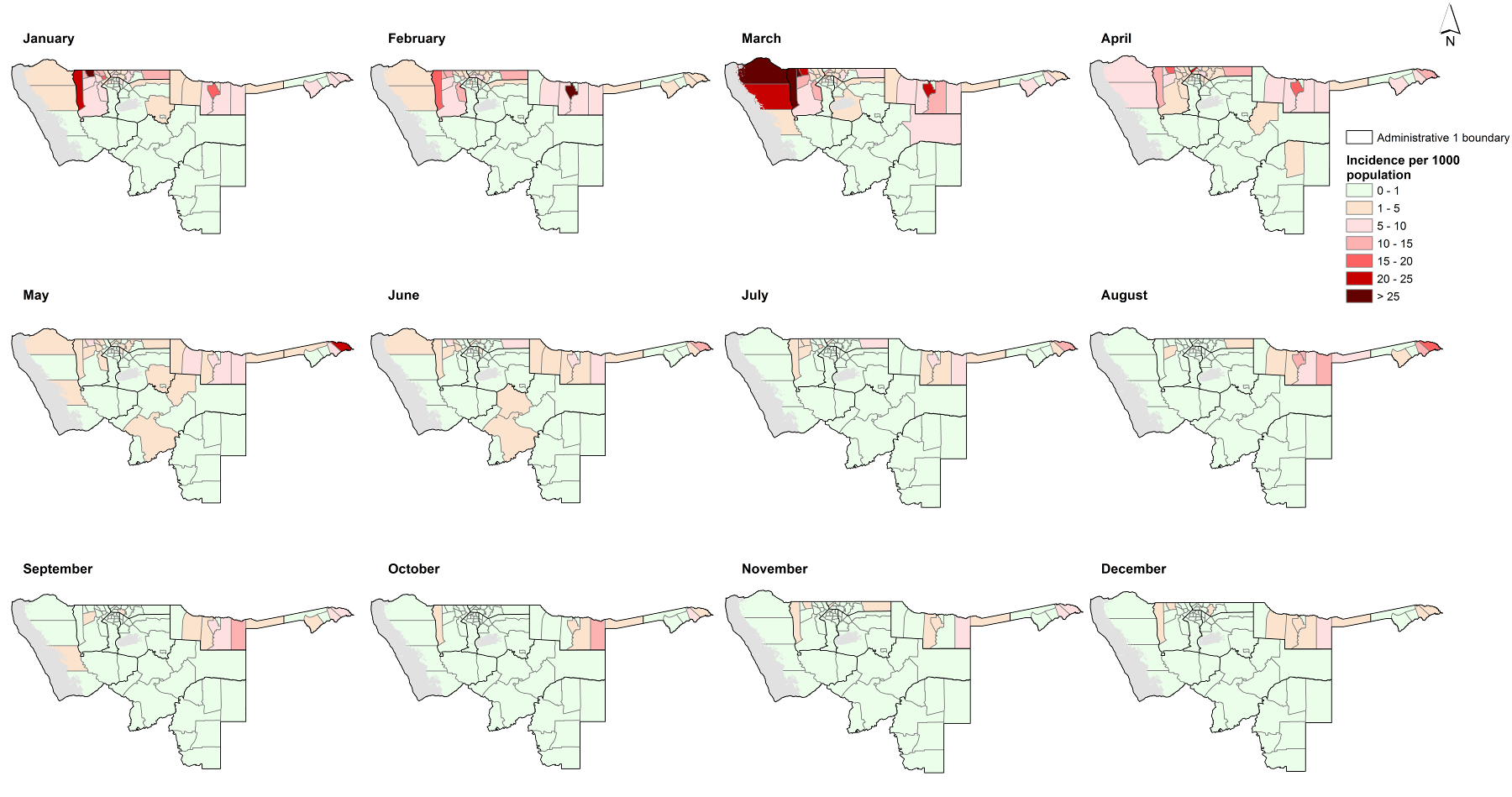
**

**Figure 1:** Map showing the average monthly malaria incidence per 1000 population at constituency level for northern Namibia in 2009 using the non-spatial method deployed by the WHO, where reported cases are adjusted for under reporting by health facilities, the slide positivity rates and facility utilisation.


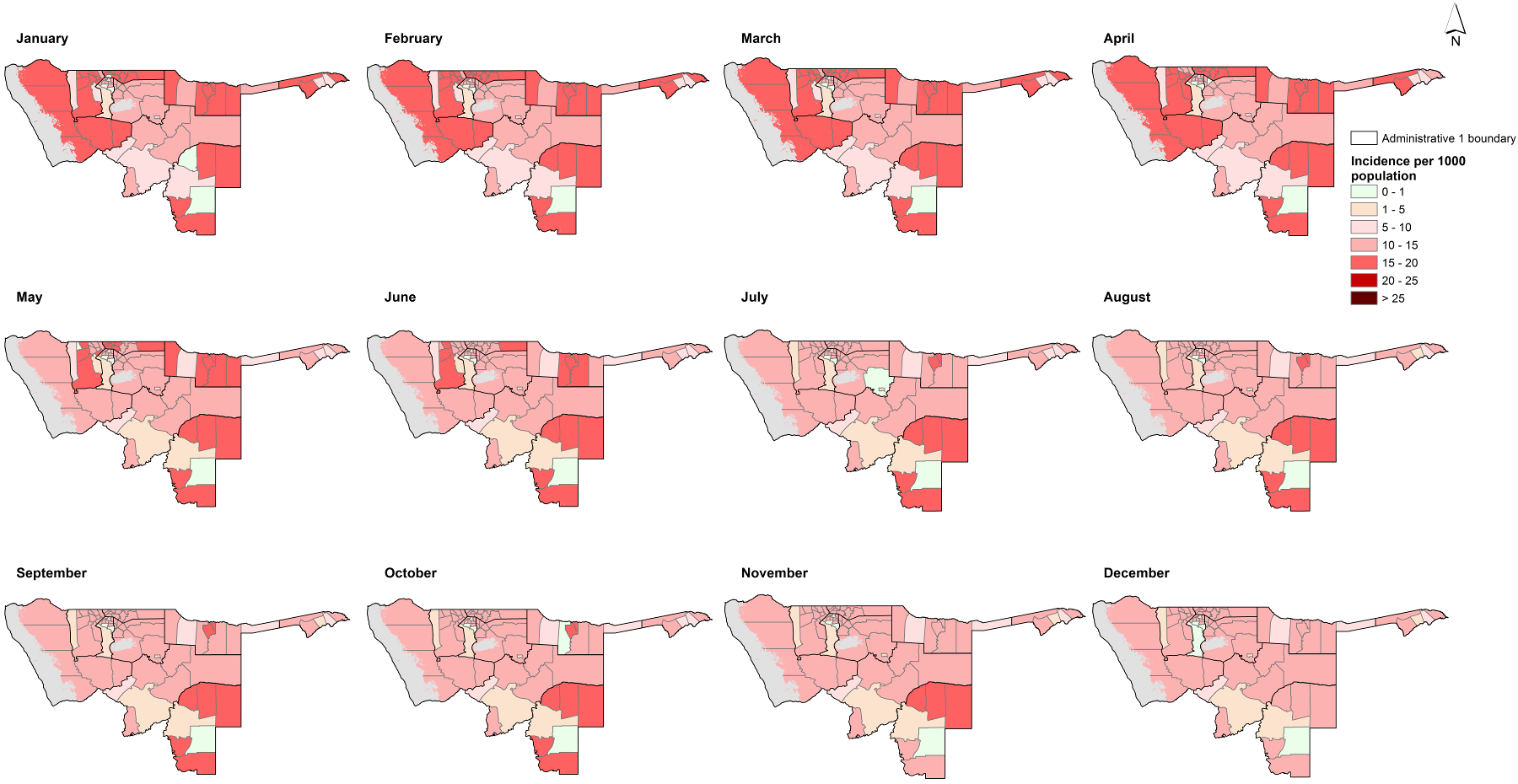


**Figure 2**: Map showing the predicted monthly malaria incidence per 1000 population at constituency level for regions in the north of Namibia in 2009 using Bayesian CAR with environmental covariates.


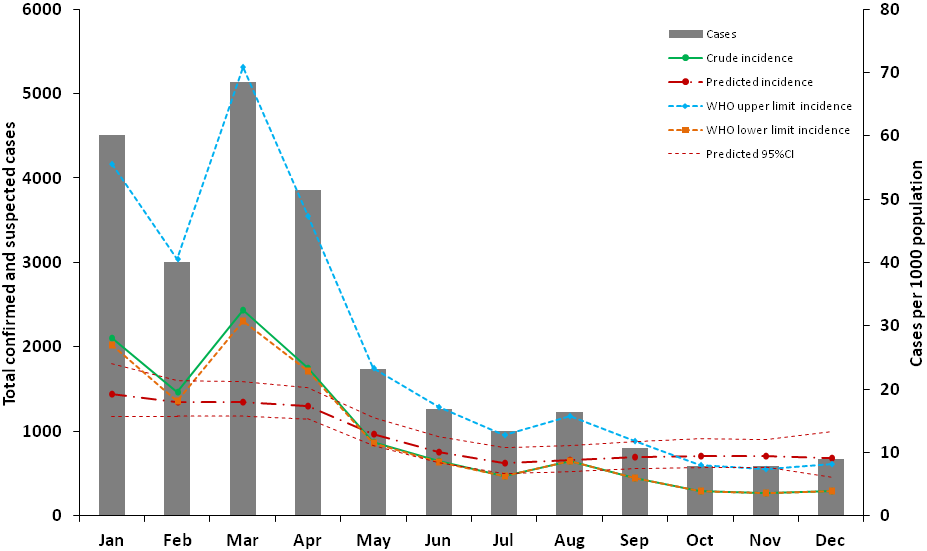


**Figure 3**: Plot of the observed cases by month in northern Namibia in 2009 (bar), the observed crude incidence (continuous black line, derived from confirmed and suspected cases combined) and the fitted incidence (dashed line) per 1000 people with associated confidence intervals.

**1.3 Comparison of Annual incidence for 2009 in northern Namibia based on WHO and Bayesian approaches**

Overall, crude annual incidence based on the parasitological and clinically diagnosed cases, but corrected for slide positivity rate, was 16 cases per 1000 population. Using the WHO approach, mean annual incidence was 23 cases per 1000 population and 13 cases per 1000 population based on the Bayesian CAR modeling approach, in the 78 constituencies in northern Namibia. Figure 4 shows maps of mean annual incidence based on both approaches. For the WHO method incidence was greater than 25 cases in some northern constituencies bordering Angola, Zambia and Botswana while from the Bayesian CAR model, the largest predictions were between 15 to 20 cases per 1000 population (Figure 4(a) and 4(b)). While risk was smoothed between adjacent constituencies based on the Bayesian method, the same was not evident in the map based on the WHO approach. Consequently, some neighbouring regions had highly contrasting risk. Concordance tests based on the annualized incidence in Ohangwena, Kavango, Caprivi and Kunene were: mean difference 5.567[CI -2.326 – 13.461] (95% limit of difference -25.542 - 36.677); Pitman’s test 0.951, *p*<0.001; Pearson’s *r* = 0.156) and in Omaheke and OtjozondJupa: mean difference 0.402[CI 0.164 – 0.989] (95% limit of difference 0.041 – 3.982) Pitman’s test 0.692, *p*<0.001; Pearson’s *r* = 0.817). These results suggested some level of concordance between the WHO approach estimates and the Bayesian predictions in the constituencies in the south of Otjozondjupa.


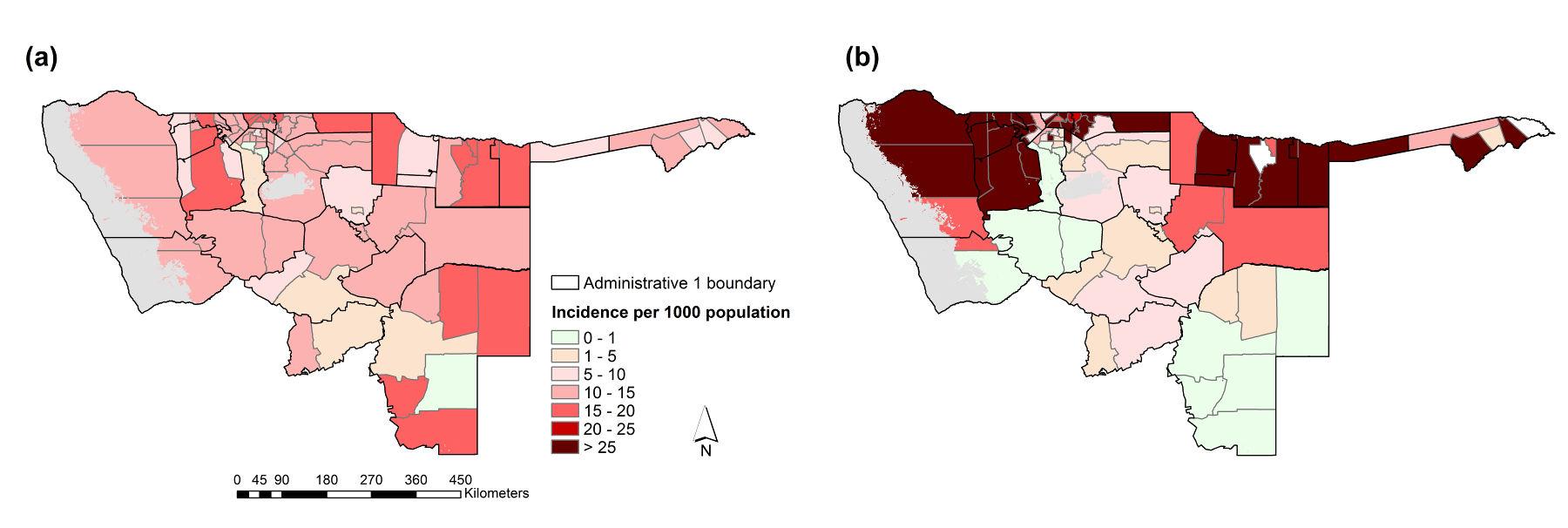


**Figure 4**: Maps showing (**a)** the mean annual incidence prediction based on Bayesian CAR with environmental covariates and **(b)** the mean incidence based on the WHO approach at constituency level in northern Namibia in 2009.

**1.4 Comparison of predicted population at risk between the WHO (non-spatial) approach and Bayesian approach**

Based on the WHO annual incidence, 806,217 (57.2%) of the population lived in areas where case incidence was greater than 15 cases per 1000 population; approximately 53,355 (3.8%) resided in regions with an average of 10 to 15 cases per 1000 population; 150,688 (10.7%) in areas with 5 to 10 cases per 1000 population; 299,830 (21.3%) in areas with greater than 1 case but less than 5 cases per 1000 population and 99,750 (7.1%) of population lived in regions with less than 1 case per 1000 population. For the Bayesian CAR model, 383,632 (27.2%) of the population lived in areas where case incidence was greater than 15 cases per 1000 population; slightly more than half 745,903 (52.9%) lived in areas where case incidence was between 10 to 15 cases per 1000 population; approximately 216,512 (15.4%) resided in regions with an average of 5 to 10 cases per 1000 population; 49,005 (3.5%) in areas with greater than 1 case, but less than 5 cases per 1000 population and 1% of population lived in regions with less than 1 case per 1000 population. The number of people estimated to live in constituencies with greater than 15 cases per 1000 population was more than double using the WHO approach compared to the Bayesian approach due to the smoothing of risk in the latter approach.

**1.5 Internal consistency check between the WHO approach and the Bayesian method**

A consistency check of the WHO formula was conducted by selecting randomly 30% of health facilities as a hold out set from the assembled list and calculation incidence using the remainder (70%). The predicted incidence, based on 70% of the data, was compared to incidence based of full (100%) of data. The objective was to evaluate the change in estimated incidence given a change in the reporting rate. The model with largest margin of difference was considered as most volatile, thus, most imprecise. The Bayesian approach was most consistent of the two methods (Table 1). The margin of difference was larger when using the WHO approach, mainly driven by the rate of reporting. This is expected, because both the numerator and denominator in the WHO approach is affected by change in actual values compared to a sophisticated predictive modelling approach that benefits from extra parameterisation.

**Table 1**: Consistency checks between WHO approach and Bayesian method

| Percentage of Data used | Average incidence per 1000 population by WHO method | Average incidence per 1000 population by Bayesian method |
| --- | --- | --- |
| 100% | 23.950 | 13.268 |
| 70% | 19.481 | 13.251 |

**1.6 Discussion**

The WHO approach resulted in higher estimates of incidence in the high transmission months in 2009 compared to the model-based approach where incidence was predicted to be higher than the crude rate only for drier low transmission months. The WHO approach is not model-based, thus, it does not benefit from spatial dependence structures introduced in full hierarchical models. This standard non-spatial approach adjusts for the rate of reporting and healthcare utilisation ignoring the spatial autocorrelation effects. Further, the assumed 100% utilisation rate (for the upper limit) is unrealistic, because, in reality other healthcare sectors (e.g. private) are often used.

The WHO method estimated incidence that exceeded 25 cases per 1000 population in some constituencies suggesting that incidence may be overestimated when using this approach (Cibulsk*is et a*l., 2011). Cibulskis et al. (2011) discussed similar findings and suggested factors such as: variation in treatment seeking patterns as a result of disproportionate cluster sampling procedures employed in the household surveys; reporting of utilisation for children under the age of five; possibility of variation in health facility use based on gender as possible sources of bias when estimating incidence. These limitations remain in the method used in this analysis. The low utilisation rates observed for the Omaheke region based on this approach may in turn be linked to under-sampling of MIS clusters rather than the actual utilisation rates which may contribute to the underestimation of incidence. True incidence, therefore, may lie close to the WHO lower limit (Figure 3) if all malaria cases are treated as a result of higher healthcare access as suggested by Cibulskis et al. (2011). In practice, it is difficult to measure utilisation precisely since healthcare access is a multidimensional phenomenon (Aday and Andersen, 1974, Ensor and Cooper, 2002). Other potential biases in incidence estimation based on this method are discussed in Cibulskis et al. (2011).

The Bayesian model yielded a mean incidence of 13 cases per 1000 population for 2009 and the monthly estimates were lower when compared to estimates based on the WHO method between January and April, but slightly higher from August to December (Figure 2 and Figure 3). These differences may be due to several factors. First, the Bayesian approach included the unstructured random component to explain unobserved effects while the inclusion of the structural effects via the GMRF introduced dependence resulting in spatial smoothing of seasonal variation (Banerjee and Gelfand, 2003, Spiegelhalt*er et a*l., 2002). Secondly, incorporating the environmental covariate explains spatial variation where data were absent in addition to providing information on the climatic suitability of malaria transmission, for example, in Omaheke region (Cra*ig et a*l., 1999, Guer*ra et a*l., 2008). The inclusion of environmental covariates may have improved the model estimates for only a few constituencies (in Kunene and Omaheke), but only marginally based on comparison of covariate and non-covariate model (in main paper).

The Bayesian approach has the advantage of addressing several sources of uncertainty that are problematic for the WHO approach. Cibulskis et al. (2011) note twelve sources of uncertainty in the WHO approach, of which three relate to reporting completeness, five to utilisation of public health facilities, and four to slide positivity rates. This study illustrates that problems related to data can be addressed sufficiently, for instance, adjusting for attendance at facility level or reporting completeness. The Bayesian CAR model was applied at facility level and, therefore, the method not only takes into account the nature of the facility, but also season and contextual environmental factors in adjusting for under-reporting. In addition, the CAR model smoothed incidence, thereby reducing the potential impacts of *mis*-reporting of cases by facilities.

**2.0 Bayesian proper model scoring rules**

There are different methods of evaluating model uncertainty. One of the proposed approach is based on the probabilistic values from predictive distribution of the model compared to actual observations (Gneiting and Raftery, 2007). A score is then said to be proper if there is consistency between the predictions and the observations (model is correctly calibrated). Gneiting and Raftery (2007) review some of these proper scoring rules, for example, the standard error score (SES), ranked probability score (RPS) and the Dawid-Sebastiani score (DSS) computed as :

$$SES(P,\begin{matrix} y \end{matrix})\begin{matrix} =\begin{matrix} (y-\mu_{P}) \end{matrix} \end{matrix}^{2}$$

$$DSS(P,\begin{matrix} y \end{matrix})\begin{matrix} =\begin{matrix} \frac{1}{2}\cdot\left(\log\left(\sigma^{2}\right)\begin{matrix} +\begin{matrix} \left(\left(y\begin{matrix} -\begin{matrix} \mu_{p} \end{matrix} \end{matrix}\right)/\sigma_{p}\right)^{2} \end{matrix} \end{matrix}\right) \end{matrix} \end{matrix}$$

$$RPS(P,\begin{matrix} y \end{matrix})\begin{matrix} =\begin{matrix} \sum_{k=0}^{\infty} \left[P\left(Y\leq k\right)-1\left(y\leq k\right)\right] \end{matrix} \end{matrix}^{2}$$

where $P$ is the predictive distribution with mean $\mu_{p}$and standard deviation$\sigma_{p}$ and $y$is the observed count (Gneiting and Raftery, 2007). The SES is comparable to square mean error $\begin{matrix} \begin{matrix} (y-\mu) \end{matrix} \end{matrix}^{2}$ except that it applies to a predictive distribution while the DSS (Dawid and Sebastiani, 1999) is an alternative measure of predictive model choice criterion (Gelfand and Ghosh, 1998). The predictive distribution was calculated for the missing data point in the input data i.e the NA. To predict a value $y_{p}$given other values, the predictive density $P(y_{p}|y_{-p})$ is given by:

$$P(y_{p}|y_{-p})\begin{matrix} =\begin{matrix} \int\pi(y \end{matrix} \end{matrix}|\theta,\begin{matrix} y_{-p} \end{matrix})\begin{matrix} \pi(\theta|y_{-p} \end{matrix})\begin{matrix} d\theta_{p} \end{matrix}$$

and obtained via a finite sum with weights $\Delta_{j}$:

$$P(y_{p}|y_{-p})\begin{matrix} \approx\begin{matrix} \sum_{1}^{j} \pi(y_{p} \end{matrix} \end{matrix}|\theta_{j},\begin{matrix} y_{-p} \end{matrix})\begin{matrix} \pi(\theta_{j} \end{matrix}|y_{-p})\begin{matrix} \Delta_{j} \end{matrix}$$

A Pearson correlation coefficient was calculated for Model 2 (M2 in the main paper) to compare the predicted values to the observed in addition to the leave one out cross validation approach. The correlation was based on 26 randomly selected health facilities in northern Namibia. Residuals based on this hold-out set were checked for spatial structure. The leave-one-out cross validation score using the conditional predictive ordinate (CPO) was evaluated in the main paper. For CPO, a prediction is validated based on the predictive distribution and the remaining data only (Cza*do et a*l., 2009, Spiegelhalt*er et a*l., 2002). Thus, it is the probability of observing a value given all other data. No data values for the fitted model failed the CPO test which is likely to happen if the approximation of the latent Gaussian Field (GF) is not sufficiently accurate (Cza*do et a*l., 2009).

**2.1 Validation Results**

Table 1 shows some of the validation results from the two Bayesian model (M1 and M2). Model comparison via DIC, SES, RPS and DSS showed that M2 was marginally better compared to M1. The standard deviation of predictive model was lower for this model compared to M1. The lower the predictive score the better the model. Figure 5 shows a scatter plot of the predicted values compared to the observed cases based on M2. This Pearson correlation coefficient for the model predictions was 0.56. A further analysis of residuals showed presence of spatial structure (Figure 6).

**Table 1**: model scoring parameters

| **Model** | **Mean deviance** | **RPS** | **SES** | **DSS** | **standard deviation of predictive distribution** | **mean of predictive distribution** |
| --- | --- | --- | --- | --- | --- | --- |
| Model 1 (without covariate) | 3461.581 | 0.6922 | 1.7039 | 0.7833 | 1.3053 | 1.1526 |
| Model 2 (with covariate) | 3460.702 | 0.6662 | 1.6093 | 0.7514 | 1.2686 | 1.1343 |

**Figure 5**: Scatter plot of the observed cases compared to the predictions for the Bayesian model with environmental covariate (M2).

**Figure 6**: semi-variogram plot showing residual autocorrelation based on Bayesian model (M2). The y-axis is the semi variance while the x-axis is distance in degree between pairs. The residuals were extracted for validation set data.

**References**

Aday, L. A. and Andersen, R. (1974) A framework for the study of access to medical care. *Health Services Research,* 9**,** 208-220.

Banerjee, S. and Gelfand, A. E. (2003) On smoothness properties of spatial processes. *Journal of Multivariate Analysis,* 84**,** 85-100.

Bland, J. M. and Altman, D. G. (1986) Statistical methods for assessing agreement between two methods of clinical measurement. *Lancet,* 1**,** 307-10.

Bland, J. M. and Altman, D. G. (2010) Statistical methods for assessing agreement between two methods of clinical measurement. *International Journal of Nursing Studies,* 47**,** 931-936.

Cibulskis, R. E., Aregawi, M., Williams, R., Otten, M. and Dye, C. (2011) Worldwide Incidence of Malaria in 2009: Estimates, Time Trends, and a Critique of Methods. *PLoS Med,* 8**,** e1001142.

Cox, N. and Steichen, T. J. (2007) CONCORD: Stata module for concordance correlation.

Craig, M. H., Snow, R. W. and Le Sueur, D. (1999) A climate-based distribution model of malaria transmission in sub-Saharan Africa. *Parasitol Today,* 15**,** 105-11.

Czado, C., Gneiting, T. and Held, L. (2009) Predictive model assessment for count data. *Biometrics,* 65**,** 1254-61.

Dawid, A. P. and Sebastiani, P. (1999) Coherent Dispersion Criteria for Optimal Experimental Design. *The Annals of Statistics,* 27**,** 65-81.

Ensor, T. and Cooper, S. (2002) Overcoming barriers to health service access: influencing the demand side. *Health Policy and Planning,* 19**,** 69-79.

Gelfand, A. E. and Ghosh, S. K. (1998) Model choice: A minimum posterior predictive loss approach. *Biometrika,* 85**,** 1-11.

Gneiting, T. and Raftery, A. E. (2007) Strictly Proper Scoring Rules, Prediction, and Estimation. *Journal of the American Statistical Association,* 102**,** 359-378.

Guerra, C. A., Gikandi, P. W., Tatem, A. J., Noor, A. M., Smith, D. L., Hay, S. I. and Snow, R. W. (2008) The limits and intensity of Plasmodium falciparum transmission: implications for malaria control and elimination worldwide. *PLoS Med,* 5**,** e38.

Ministry of Health and Social Services (2008) Namibia Demographic and Health Survey, 2006-07. Windhoek, Namibia, Ministry of Health and Social Services.

Ministry of Health and Social Services (2010) Namibia malaria indicator survey 2009. Windhoek.

Pitman, E. J. G. (1939) A Note on Normal Correlation. *Biometrika,* 31**,** 9-12.

Spiegelhalter, D. J., Best, N. G., Carlin, B. P. and Van Der Linde, A. (2002) Bayesian measures of model complexity and fit. *Journal of the Royal Statistical Society: Series B (Statistical Methodology),* 64**,** 583-639.

Unicef (2012) Childinfo: Monitoring the situation of women and children. *Multiple Indicator Cluster Surveys.*
